# The ARGO dataset: Annotated and delineated intracardiac electrograms of post-ischemic ventricular tachycardia

**DOI:** 10.1371/journal.pone.0350993

**Published:** 2026-06-15

**Authors:** Marco Orrù, Giulia Baldazzi, Davide Zirolia, Livio Bertagnolli, Graziana Viola, Maria Giuliana Solinas, Danilo Pani

**Affiliations:** 1 Department of Informatics, Bioengineering, Robotics and Systems Engineering, University of Genova, Genova, Italy; 2 MeDSP Lab, Department of Electrical and Electronic Engineering, University of Cagliari, Cagliari, Italy; 3 Clinical and Interventional Cardiology Unit, Santissima Annunziata Hospital Sassari, Sassari, Italy; 4 Electrophysiology and Cardiac Pacing Unit, San Maurizio Regional Hospital, Bolzano, Italy; 5 Cardiology Unit (UTIC), Santissima Trinità Hospital, Cagliari, Italy; 6 Department of Biomedical Sciences, University of Sassari, Sassari, Italy; Policlinico Casilino, ITALY

## Abstract

The identification of abnormal ventricular potentials (AVPs) in intracardiac electrograms of post-ischemic ventricular tachycardia patients is challenging, and the development of automated tools is hampered by the lack of annotated benchmarking datasets. This work presents the ARGO dataset, the first open-access collection of manually annotated left-ventricle post-ischemic ventricular tachycardia electrograms. The dataset includes 1962 signals from nine patients, including unipolar and bipolar electrograms, 12-lead surface ECGs, and the local activation time and voltage maps, with annotations of the bipolar EGMs in terms of their nature (i.e., Physiological, AVP, or Unknown) and the delineation of the onset/end of the AVPs. The annotation is the result of three independent annotations by as many expert electrophysiologists, including a final consensus, for enhanced reliability and accuracy of the dataset. Leveraging good inter-rater and intra-rater reliability, the proposed validation analysis also provides quantitative estimates of the tolerance associated with AVP delineation error. Even considering the limitations of the proposed dataset affecting generalizability, ARGO provides a unique resource to support the development and validation of computational tools for AVP identification and characterization.

## Introduction

Intracardiac electrograms (EGMs) analysis is crucial for guiding radiofrequency catheter ablation (RFCA) [1] in the treatment of several cardiac arrhythmias, especially post-ischemic ventricular tachycardia (VT). For the latter, arrhythmogenic areas are commonly identified during electroanatomic (EA) mapping by the presence of abnormal ventricular potentials (AVPs), characterized by late activations with high-frequency fractionated components in local bipolar EGMs [2,3]. Nonetheless, AVP identification is a time-consuming and error-prone process. In the scientific literature, several studies [4–10] explored computational methods leveraging signal processing techniques often combined with artificial intelligence to assist clinicians in this task. However, the development and validation of such algorithms heavily rely on the availability of benchmarking datasets.

In this context, publicly available datasets related to intracardiac recordings are rare and not focused on VT arrhythmogenic sites, thus posing significant obstacles to research in this field. In this regard, the only publicly available dataset is the Intracardiac Atrial Fibrillation Database [11], which focuses on atrial fibrillation and flutter. Conversely, other works present datasets with indications of atrial activity onset/end in different types of arrhythmias [12] or propose datasets encompassing synchronized surface and intracardiac signals for the detection of various arrhythmias (including VT) as well as for the identification of AVPs [13,14], but free and public access is not guaranteed.

On the other hand, several annotated datasets have been published over the past decades [15], but limitedly to 12-lead surface ECG signals. For instance, the SPH Dataset [16], the MIT-BIH [17], the INCART [18], and the PTB-XL [19] provide repositories of labeled 12-lead surface ECG signals to explore and analyze different arrhythmias and cardiac conditions. Other datasets proposed in the literature, e.g., the Shaoxing People’s Hospital Dataset [20], the European ST-T Database [21], and LUDB [22], beyond the signal class labels, provide detailed annotations of the main 12-lead surface ECG waves, as their delineation.

As a result, there is an urgent need to establish and share open datasets in invasive cardiac electrophysiology, and especially datasets of post-ischemic VT EGMs, to foster bioengineering research in the field and support the development and validation of signal processing, delineation, feature extraction, and automatic classification methods for the study and recognition of arrhythmogenic sites.

This work addresses this challenge by introducing the ARGO dataset, the first open-access dataset of post-ischemic VT EA recordings from the left ventricle (LV) with the CARTO^®^3V6 system. A preliminary version of the dataset, with limited features (i.e., majority voting for final annotation) was presented in a previous study [23]. The final version of the ARGO dataset significantly improves the preliminary one, as it features synchronized electrophysiological recordings (bipolar and unipolar EGMs together with 12-lead ECGs), information on both catheter type and electrode pairs, electro-anatomical maps (i.e., local activation time (LAT) and voltage maps), and detailed information on the spatial localization of the ablation targets. Moreover, it provides expert-consensus annotations of bipolar EGMs (i.e., physiological, AVP, or unknown) and their delineation, reporting the onset and end of pathological activation in AVPs. The reliability and consistency of these annotations were confirmed through robust statistical validation.

The ARGO dataset is the first free public dataset of this kind, embodying a valuable resource for the development and benchmarking of AVP detection and delineation algorithms. To ensure its widespread diffusion, it is available on PhysioNet [24] at the following link: https://doi.org/10.13026/afxd-9424, and its main features are detailed in the following sections.

## Methods

### Study population

The retrospective observational study behind the dataset creation was approved by the Independent Ethics Committee of the Azienda Tutela Salute, Sardegna (Prot. n. 351/2021/CE, date of approval: 13/07/2021) and performed following the principles outlined in the 1975 Helsinki Declaration, as revised in 2000. Participant recruitment and data access were conducted retrospectively (data access period: 15/07/2021–01/09/2021). Nine participants (age: 66 ± 10 years old 78% males, ejection fraction: 29.4% ± 5.8%, mean ± standard deviation) affected by post-ischemic VT who underwent LV EA mapping and RFCA between 2017 and 2018 at the San Francesco Hospital (Nuoro, Italy) were enrolled in the study. They provided their written informed consent; all identifying information that could link data to a given participant has been removed by anonymization, clearing both direct and indirect identifiers. Access to identifiable participant information during and after data collection was restricted to a single author (G.V.).

### Clinical data collection

Data acquisition was performed during routine electrophysiological studies and RFCA procedures on post-ischemic VT patients, as detailed in Supplementary Material, section I.A (S1 File).

During pre-ablation EA mapping, all spatial data of the LV geometry, EGMs and the 12-lead surface ECGs were acquired simultaneously. All clinical procedures were performed by the same electrophysiologist (G.V.). It is noteworthy that all the included EGMs and related EA maps were collected solely under sinus rhythm, ensuring physiological consistency across all cases and strengthening the reliability of annotation procedure. Under these conditions, the recorded EGMs primarily reflect intrinsic activation wavefront propagation, without the potential confounding effects introduced by external paced activations. In particular, pacing may alter local activation patterns and introduce variability related to the relative spatial orientation between the pacing electrode and the recording dipole, which could bias EGM morphology and retrospective interpretation. By restricting data collection to sinus rhythm, such sources of variability were minimized. Remarkably, this approach reflects one of the standard mapping strategies used in clinical practice for substrate characterization.

The CARTO^®^3V6 system (Biosense Webster, Inc., Diamond Bar, California) was used for all the procedures. Intracardiac EGMs and EA information provided in this dataset were recorded using PentaRay 2-6-2 mm, ThermoCool SmartTouch and ThermoCool SmartTouch SF (Biosense Webster, Inc., Diamond Bar, California), specifically, unipolar and bipolar EGMs, the 12-lead surface ECG, and voltage and LAT EA maps. The adopted mapping system enables the acquisition of reliable EGMs when the catheter was in contact with the endocardium. To ensure this, a short-term 2.5-s trace was recorded for every mapped point, but the annotation of the dataset is referred to the reliable beat located around the last cardiac cycle, according to the CARTO^®^3V6 system, which is identified on the basis of the ventricular activity.

Both 12-lead ECG and EGMs were sampled at 1 kHz, with resolution of 0.003 mV/LSB. All the recordings underwent band-pass filtering by the CARTO^®^3V6 system: bipolar EGMs were filtered in the 16 ÷ 500 Hz band, unipolar EGMs in the 2 ÷ 240 Hz band, and 12-lead ECG traces in the 0.5 ÷ 120 Hz band. Specifically, these are the standard settings routinely employed during the clinical procedures and were configured by the medical team. These parameters were uniformly employed across all procedures, and no additional offline filtering was applied. The same acquisition and filtering parameters were consistently adopted across all nine procedures, ensuring uniformity and comparability, and reflecting real-world clinical practice.

### Electrophysiological data annotation

All electrophysiological data, collected before the RFCA was performed, were exported from the CARTO^®^3V6 system and anonymized.

Among all the recorded signals, 1962 bipolar EGM segments were manually and independently annotated and delineated by three experienced electrophysiologists (hereafter named Annotator1, Annotator2, and Annotator3) using a custom graphical user interface, as detailed in Supplementary Materials, Section I.B (S1 File), blindly with respect to the patient. The annotators were asked to label each bipolar EGM segment as “*Physiological*”, “*AVP*”, or “*Unknown*”. Specifically: the Physiological class encompassed all EGMs exhibiting typical physiological patterns, thus reflecting normal electrophysiological activity of the LV endocardium; the AVP class includes those EGMs that exhibit abnormal ventricular patterns, indicating arrhythmogenic areas; the Unknown class included all EGMs for which a confident assignment to either the Physiological or AVP class was not possible. Specifically, this class comprised EGMs affected by ambiguous morphology or acquisition-related uncertainty, including extra-systolic beats, very low-voltage recordings, EGMs in proximity to valvular structures, atrial or His bundle components.

Although multiple definitions of abnormal potentials have been proposed, no universally accepted criteria currently exist for their identification. Consequently, AVP annotation in clinical practice commonly relies on the recognition of characteristic EGM morphologies rather than strictly predefined quantitative rules. In line with the literature [3], abnormal ventricular activity is commonly characterized through several EGM morphologies, including: late potentials (LPs) occurring after the termination of the surface QRS complex, either as continuous fragmented activity or as isolated components, without applying a specific voltage cutoff [25]; early potentials (EPs) represented by fragmented EGMs inscribed within the QRS complex and typically recorded in border zones [25]; local abnormal ventricular activities (LAVAs) defined as sharp, high-frequency ventricular signals distinct from the far-field ventricular EGM and often occurring during or after the QRS complex [2]; and fractionated electrograms, typically characterized by low amplitude, prolonged duration, and multiple fragmented components reflecting slow and heterogeneous conduction within scar tissue [26]. Accordingly, AVPs in the present dataset were identified based on these morphological characteristics taken from the literature, together with the clinical interpretation of the electrophysiologists performing signal annotation. In the case of an EGM labelled as AVP, the annotators were additionally asked to delineate its onset and end.

It is worth noting that the bipolar EGMs labelled as AVPs do not necessarily represent target ablation points of the electrophysiology procedure, as the study is retrospective (so a given point could have been overlooked during the procedure) and different ablation strategies could have been deployed to treat the patient.

**Fig 1:**
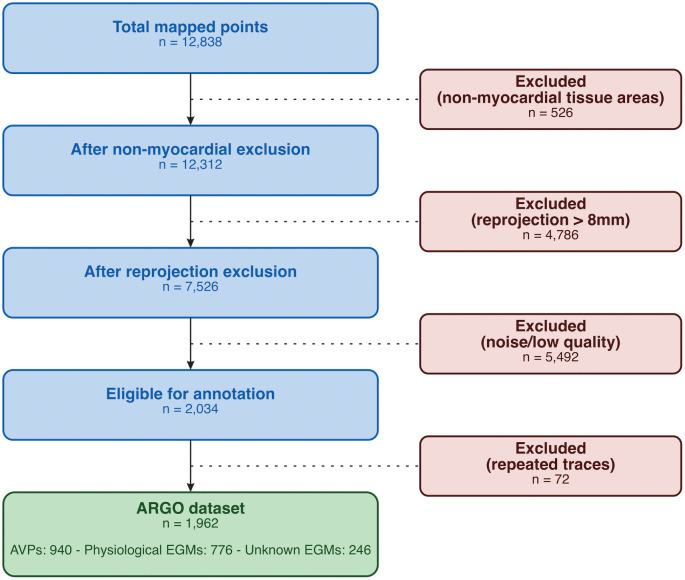
Pipeline for ARGO construction and exclusion criteria. Flow diagram illustrating the selection and filtering process used to build the ARGO dataset.

Although the electrophysiological procedures originating the dataset included a larger number of mapped points than annotated ones, several points were excluded: in particular, all EGMs recorded in non-myocardial tissue areas, such as the mitral and aortic valves, and all EGMs associated with EA points that were spatially located onto the LV with a projection distance greater than 8 mm (to include only EGMs with a reliable localization on the map). The remaining signals were subsequently reviewed for signal quality by visual inspection by an experienced electrophysiologist. EGMs presenting severe interference, poor catheter contact, or signal conditions hampering reliable annotation were excluded. Importantly, noise-related exclusions were not based on predefined quantitative thresholds but on expert assessment of signal interpretability. A detailed summary of the selection pipeline and of the exclusions applied at each step is provided in Fig 1.

Considering these exclusion criteria, a total of 1962 EGMs were annotated and included in the ARGO dataset. Table 1 reports the number of EGMs per participant.

Notably, to carry out an intra-annotator variability analysis, a subset of 209 signals was shown three times to the annotators. Therefore, every annotator was asked to label and delineate a total of 2380 signals.

**Table 1:** Number of EGMs annotated for each patient and included in the ARGO dataset.

Patient ID	Pt1	Pt2	Pt3	Pt4	Pt5	Pt6	Pt7	Pt8	Pt9
# EGMs	157	104	90	471	46	839	76	129	50

### Expert consensus

A final consensus among the annotators was undertaken to enhance annotation reliability [27], leveraging individual (but anonymized) annotations previously provided by the three annotators. Consequently, for each EGM, a definitive consensus-driven annotation was derived and included in the ARGO dataset, along with the three individual annotations obtained in the first independent annotation procedure. To rigorously minimize potential biases, in particular confirmation and dominance biases, several safeguards were implemented during the expert consensus. In particular, the consensus sessions took place after a sufficiently long interval following the individual annotations and were guided by a uniquely designated moderator (i.e., a bioengineer who did not participate in the initial annotation phase), whose role was to facilitate the discussion without influencing clinical judgment and without any clinical hierarchy over the experts. This precaution ensured that the discussion remained focused on objective signal morphology rather than professional seniority. Furthermore, all independent annotations were anonymized within a graphical user interface using neutral, color-coded markers. This choice ensured that the experts reached a unanimous decision based strictly on signal morphology rather than the identity or perceived expertise of the original annotator. Finally, no majority-rule system was leveraged: for each EGM, the annotators were required to discuss and reach a unanimous agreement on the class (AVP, Physiological, or Unknown). If the AVP class was assigned, they collectively finalized the delineation by either selecting from the previous onset/end points or identifying new temporal fiducial locations. This “veto power” ensured that even the most conservative opinions were fully discussed and resolved, effectively limiting dominance effects.

The expert consensus required 13 days for a total of about 26 working hours, and was carried out using an ad-hoc interface developed in MATLAB, as detailed in Supplementary Material, Section I.B (S1 File).

At the end of the consensus, the ARGO dataset consists of 776 physiological potentials, 940 AVPs, and 246 unclassifiable EGMs. Table 2 reports total number of EGMs and the numerosity of AVPs, physiological and unknown EGMs for each patient.

**Table 2:** Distribution of EGM categories across the nine patients obtained with the consensus. The table reports the number of annotated AVPs, physiological and unknown EGMs for each patient, along with the total number of EGMs per patient and across the entire dataset.

EGM class	Patient									Total
**1**	**2**	**3**	**4**	**5**	**6**	**7**	**8**	**9**
AVPs	85	52	67	74	21	508	34	66	33	940
Physiological EGMs	54	36	17	386	12	168	35	54	14	776
Unknown EGMs	18	16	6	11	13	163	7	9	3	246
Total	157	104	90	471	46	839	76	129	50	1962

The interested reader can find the description of the released dataset structure and organization in Supplementary Material, Section II (S1 File), according to the PhysioNet requirements.

## Technical validation

A rigorous statistical validation process was conducted to ensure the reliability and consistency [28] of the ARGO dataset annotations. The validation was based on the annotations derived from the three experts independently and their consensus. The approaches adopted to assess the reliability and the consistency both in class annotation and AVP delineation are described in the following sections separately. All statistical analyses were performed using STATA 18 (StataCorp. 2023. Statistical software. StataCorp LLC.).

### Validation analysis for class annotation

To evaluate the consistency between the three experts (i.e., raters) in performing the class annotation, an inter-rater reliability analysis was conducted by Fleiss’ κ statistics [29] on the full dataset (i.e., 1962 EGMs).

The reliability of each rater was investigated through an intra-rater test-retest analysis [30], by Fleiss’ κ, on a repeated subset of the data (i.e., 209 EGMs), which were labelled and delineated during three different independent sessions.

Additionally, the consistency of each rater against the final consensus was evaluated using Cohen’s κ statistics [31]. According to the Landis and Koch [32] scale, agreement levels were categorized as follows: < 0.00 poor; 0.00–0.20 slight; 0.21–0.40 fair; 0.41–0.60 moderate; 0.61–0.80 substantial; 0.81–1.00 almost perfect.

As reported in Table 3, the inter-rater agreement reached 0.61, placing it at the lower boundary of the substantial level. Indeed, the 95% CI (0.58–0.63) overlapped with the moderate range. While this highlighted the intrinsic variability between raters and the complexity of achieving uniform annotation in bipolar EGMs, the statistical precision (narrow CIs) and the high intra-rater reliability (up to 0.79) confirmed the stability of the experts’ judgement.

**Table 3:** Summary of reliability in class annotation. Fleiss’ κ was used for overall inter-annotator and intra-annotator reliability and Cohen’s κ for individual versus consensus reliability.

Analysis	Fleiss’ κ	95% CIs
Inter-annotators	0.61	[0.58;0.63]
Intra Annotator1	0.76	[0.70;0.82]
Intra Annotator2	0.75	[0.69;0.81]
Intra Annotator3	0.79	[0.74;0.85]
	**Cohen’s** κ	**95% CIs**
Annotator1 vs Consensus	0.58	[0.55;0.61]
Annotator2 vs Consensus	0.76	[0.74;0.79]
Annotator3 vs Consensus	0.78	[0.75;0.80]

Finally, the Cohen’s κ showed a substantial agreement between Annotator2 and Annotator3 (i.e., 0.76 and 0.78, respectively) with the Consensus, and a moderate agreement for the Annotator1 (i.e., 0.58). Representative disagreement cases, which help contextualize these results, are presented in Supplementary Material, Section III.C (S1 File).

**Fig 2:**
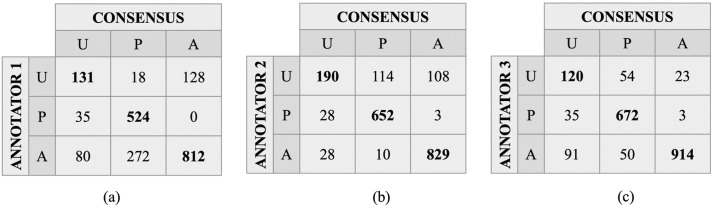
Contingency tables comparing the class annotations independently provided by each rater against the class annotations defined during the consensus meetings. From left to right, the contingency tables that compare the classification of Annotator1, Annotator2 and Annotator3 against the consensus across the categories AVP (A), Physiological (P) and Unknown (U). In each table, the number of EGMs that were classified coherently by both the annotator and the consensus (out of the 1962 labelled EGMs) was highlighted in bold.

The contingency tables reported in Fig 2 compared the annotations independently provided by each rater against the final consensus. From such results, a different ability in the recognition of the different EGM classes emerged across the three experts: indeed, Annotator 3 showed a higher proficiency in identifying definitive physiological EGMs and AVPs, closely aligning with the collective consensus. Such findings further confirmed the importance of involving multiple raters for a more reliable evaluation of the bipolar EGMs nature.

### Validation analysis for AVP delineation

The validation analysis for AVP delineation leveraged the intraclass correlation coefficient (ICC) [33], computed using the single-measure two-way mixed-effect model (i.e., ICC(3,1)), with 95% CIs. This analysis was structured into three distinct tracks:

• Inter-rater reliability, assessed on the unrepeated dataset (1962 EGMs) for all instances where all the three raters independently assigned the AVP class, using the absolute agreement to evaluate the extent to which annotators provided interchangeable AVP delineations.
• Intra-rater reliability, estimated on the repeated subset for EGMs where the rater consistently chose the AVP class across all three sessions, using consistency of agreement to quantify the reproducibility of annotations from the same expert, independently from possible systematic differences.
• Consensus agreement analysis, evaluated by considering only the EGMs for which the individual rater’s classification agreed with the final consensus in assigning the AVP class, using absolute agreement to evaluate each annotator’s accuracy with respect to the reference standard.

The 95% CIs of the ICC estimate were used to determine the level of reliability in this analysis, according to the benchmarks proposed by Koo and Li [34] (i.e., < 0.5 poor reliability; 0.5–0.75 moderate reliability; 0.75–0.9 good reliability; > 0.90 excellent reliability).

As shown in Table 4, the inter-rater analysis revealed good reliability for AVP onset delineation (ICC: 0.87; CI: 0.83–0.90) and moderate to excellent reliability AVP end delineation (ICC: 0.89; CI: 0.74; 0.94). These results highlighted a strong level of agreement among the raters in determining both AVP boundaries, and possibly an intrinsic common set of criteria for AVP delineation. Similarly, high metrics were observed in the intra-rater reliability and in the consensus agreement analyses, demonstrating a high precision and stability of experts’ AVP delineation markings.

**Table 4:** Summary of reliability in AVP delineation. ICC(3,1) was employed to assess inter-rater, intra-rater, and individual raters against consensus agreement.

Analysis	Onset		End	
**ICC**	**95% CIs**	**ICC**	**95% CIs**
Inter-annotators	0.87	[0.83;0.90]	0.89	[0.74;0.94]
Intra Annotator1	0.94	[0.92;0.96]	0.97	[0.96;0.98]
Intra Annotator2	0.92	[0.88;0.94]	0.97	[0.95;0.98]
Intra Annotator3	0.90	[0.87;0.93]	0.96	[0.95;0.97]
Annotator1 vs Consensus	0.84	[0.77;0.88]	0.89	[0.58;0.95]
Annotator2 vs Consensus	0.89	[0.87;0.90]	0.94	[0.93;0.95]
Annotator3 vs Consensus	0.89	[0.88;0.91]	0.97	[0.96;0.97]

Notably, ICC values for the AVP end were higher than those for the onset, across all scenarios. This discrepancy likely reflects the intrinsic difficulty of the AVP onset delineation, due to the potential overlapping far-field components, which can obscure the precise beginning of local activation. In contrast, since the AVP end seldom overlaps with other EGM components, its delineation was not hindered.

Finally, an error tolerance in AVP onset and end delineation was estimated in terms of the mean and standard deviation of the non-absolute differences, or errors (in ms), between median delineator (i.e., the median of the individual delineations) and the consensus, following the recommendations of the CSE Working Party [35]. Applying a *strict* *criterion* (μ ± σ), the delineation error was 4 ± 18 ms for the AVP onset and 1 ± 9 ms for AVP end. However, as stated by Martínez et al. [36], some authors might prefer a *loose* *criterion* (μ ± 2σ). Under this one, the delineation error tolerances correspond to 4 ± 36 ms and −1 ± 18 ms for AVP onset and end, respectively. When considering absolute errors (i.e., in case only the magnitude of the error is relevant) the tolerances correspond to 8 ± 16 ms (μ ± σ) for the onset delineation and 5 ± 7 ms (μ ± σ) for the end delineation.

### Electrophysiological characterization

An additional descriptive characterization was performed to better define the electrophysiological properties of the dataset presented in this work. This characterization focused on the distribution of bipolar EGM peak-to-peak amplitudes across the three annotated classes, together with the analysis of AVP duration. It is important to note that these EGM characteristics were not employed as annotation criteria, rather, the analysis is reported to offer additional insights regarding the electrophysiological characteristics of the signals included in the dataset.

**Fig 3:**
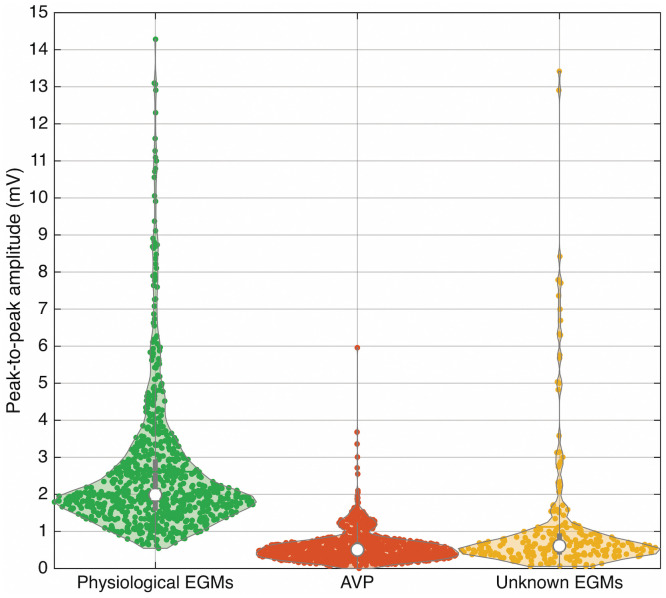
Distribution of bipolar EGM peak-to-peak amplitudes across annotated classes. From left to right, violin plots of bipolar EGM peak-to-peak amplitudes for EGMs annotated as Physiological (green), AVP (red), and Unknown (yellow).

Since peak-to-peak amplitude is a parameter widely used during substrate-guided ablation procedures, offering an indirect measure of tissue integrity and the degree of electrophysiological alteration, its distribution across the three annotated classes is proposed. As illustrated in Fig 3, physiological EGMs tended to show higher amplitudes with broader variability, while AVPs were predominantly associated with lower amplitudes, consistently with the characteristics typically observed in scar-related substrates. EGMs labelled as unknown, given their ambiguous nature, showed intermediate and more heterogeneous amplitude distributions.

**Fig 4:**
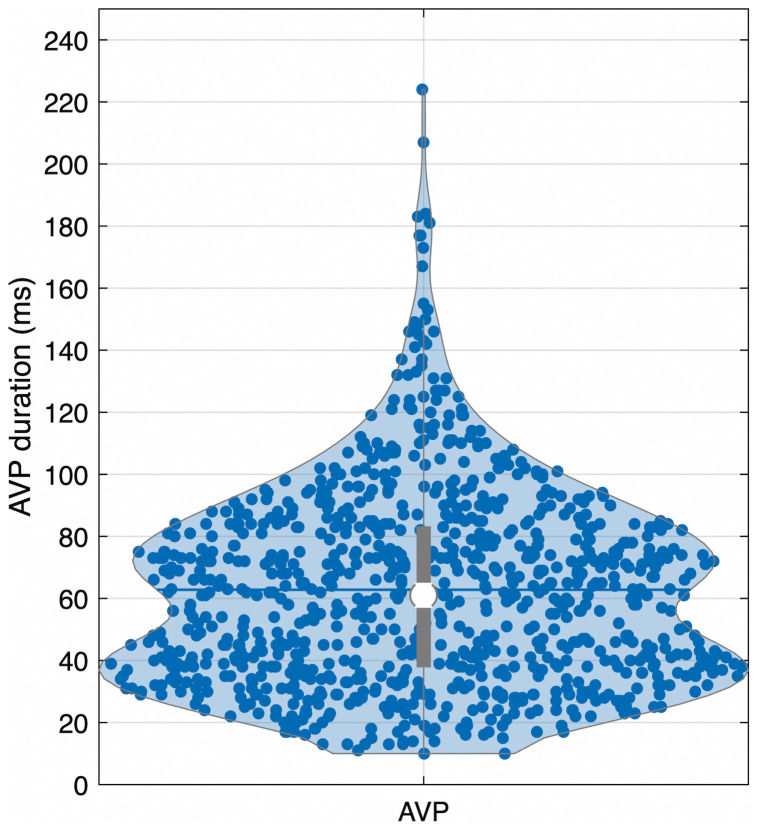
Distribution of AVP duration of annotated AVPs.

Moreover, the duration of the pathological component was characterized based on the final consensus delineations of AVP onset and end. Specifically, AVP duration was defined as the interval between the annotated onset and end points. As shown in Fig 4, the resulting distribution was characterized by a median duration of 61 ms and an interquartile range of 45 ms.

Additional details on dataset characterization are provided in the Supplementary Materials, Section III (S1 File).

### Study limitations

The authors acknowledge several limitations that should be considered when using the ARGO dataset.

First, the dataset is primarily intended to support the development and validation of computational algorithms; therefore, it should not be used to derive clinical conclusions. This limitation arises from the relatively small cohort size, both in terms of the number of patients and the number of EGMs available per patient. The number of patients is constrained by the limited frequency of this kind of procedure, whereas the number of EGMs per patients by the selection of good-quality EGMs for reliable manual annotation and delineation. Remarkably, the presence of manual and independent annotations by three clinical experts and their consensus over any single EGM makes the whole process difficult to scale to substantially larger cohorts. Overall, the limited number of patients, along with uneven distributions of EGMs and related classes across participants, restrict the representativeness and generalizability of the dataset, especially in terms of scar patterns.

Another limitation is the absence of metadata on pharmacological therapy: despite all patients underwent antiarrhythmic drug therapy before the procedure and were refractory to pharmacological treatment, as typically required for VT ablation candidates, detailed information on drug regimens was not available.

Additionally, as this is a retrospective study, the ablation targets were determined during the procedures based solely on the clinical information available at that time, without any input from the proposed annotation. Consequently, EGMs annotated as AVPs do not necessarily correspond to the sites ultimately targeted for ablation. Furthermore, different ablation strategies were employed according to the clinical practice and operator judgement at the time of the procedures (e.g., core isolation and other approaches). For these reasons, a direct comparison between AVP annotations and ablation targets would be difficult to interpret and would not support robust clinical conclusions. Procedural and follow-up outcome data were also not collected within the current dataset.

A further limitation concerns the limited clinical and technological heterogeneity. All procedures were performed in a single center by a single operator using a single mapping strategy and a single EAM system. In particular, the use of a single recording system (i.e., the CARTO^®^3V6 system) with a limited set of catheters limits dataset generalizability to centres using alternative EAM systems (e.g., EnSite or Rhythmia) or multipolar catheter technologies. Although the use of a uniform acquisition improves technical consistency in terms of bioamplifier characteristics and analog-to-digital conversion, it also limits the diversity of recorded signals. Remarkably, this aspect does not substantially compromise the observed EGM morphologies, as the catheters used in this study (PentaRay and ThermoCool SmartTouch) feature conventional inter-electrode spacing commonly adopted in the clinical practice, but the use of catheters with different inter-electrode spacing may have resulted in variations in EGM morphology and amplitude [37]. In addition, the adopted filtering parameters may have influenced the observed EGM morphologies [37]. Nevertheless, the signals provided in the ARGO dataset correspond exactly to those used during the clinical electrophysiological studies performed in the hospital that collected the signals, and therefore share the same limitations and reflect the the real-world acquisition conditions that routinely guide post-ischemic VT substrate analysis and the identification of arrhythmogenic sites for subsequent ablation.

A last limitation relates to the annotation process. Although confirmation and dominance biases were mitigated through the adoption of several safeguards, as described in the Expert Consensus section, it cannot be entirely excluded that some degree of bias may have been introduced by dominance effects in the consensus discussion, potentially influenced by more assertive annotators.

## Conclusions

The ARGO dataset addresses a substantial gap in the literature, namely the lack of publicly available datasets of intracardiac electrograms from post-ischemic VT patients with expert annotations. Its primary objective is to provide a consistently annotated resource to support methodological and computational research on arrhythmogenic substrates in post-ischemic VT, offering quantitative information for AVP classification and delineation, as well as reference estimates for human-like AVP delineation error tolerance. The presented dataset is freely provided under the Creative Commons Attribution-NonCommercial-ShareAlike 4.0 International (CC BY-NC-SA 4.0) license can be used with proper citation to this document, and can be freely downloaded from PhysioNet.

## Supporting information

S1 File Supplementary material for the article. (DOCX)
