# Supplementary material for: The ARGO dataset: Annotated and delineated intracardiac electrograms of post-ischemic ventricular tachycardia
Source: PLoS One. 2026 Jun 15;21(6):e0350993. doi: 10.1371/journal.pone.0350993 (PMC13268154; doi:10.1371/journal.pone.0350993)
Supplement: S1 File — (DOCX) [file pone.0350993.s001.docx]

Supplementary Material of the article

**The ARGO Dataset: annotated and delineated intracardiac electrograms of post-ischemic ventricular tachycardia**

Marco Orrù^1,2^, Giulia Baldazzi^2^, Davide Zirolia^3^, Livio Bertagnolli^4^, Graziana Viola^5^, Maria Giuliana Solinas^6^, and Danilo Pani^2^

^1^*Department of Informatics, Bioengineering, Robotics and Systems Engineering (DIBRIS), University of Genoa, Genoa, Italy*

^2^*Department of Electrical and Electronic Engineering (DIEE), University of Cagliari, Cagliari, Italy*

^3^*Clinical and Interventional Cardiology Unit, Santissima Annunziata Hospital, Sassari, Italy*

^4^*Electrophysiology and Cardiac Pacing Unit, San Maurizio Regional Hospital, Bolzano, Italy*

^5^*Cardiology Unit (UTIC), Santissima Trinità Hospital, Cagliari, Italy*

^6^*Department of Biomedical Sciences, University of Sassari, Sassari, Italy*

*^*^Corresponding author:*

Giulia Baldazzi

giulia.baldazzi@unica.it

# Methods

## Clinical procedures for data collection

Data collection followed routine clinical procedures for electroanatomical (EA) mapping and subsequent radiofrequency ablation (RFCA) for post-ischemic ventricular tachycardia (VT) patients. Specifically, according to the clinical practice, patients were monitored by 12-lead surface ECG and continuous invasive blood pressure from radial artery. After a deep conscious sedation, a quadripolar catheter and an intracardiac echocardiography catheter were firstly placed into the right ventricle through femoral vein access. Then, an echo-guided trans-septal puncture was performed, and a multi-electrode mapping catheter was then placed inside the left ventricle (LV). In this phase, an intravenous bolus of heparin was administrated to the patient according to the patient’s body weight. Once the catheters were correctly placed, programmed ventricular stimulation was performed for the induction of VT or any other ventricular arrhythmia (VA) before EA mapping, to define the induction protocol to be used after ablation; if VA was inducible, sinus rhythm was restored by electrical cardioversion. Then, LV substrate-guided mapping was performed, during which electrograms (EGMs) were collected in sinus rhythm. This adopted mapping strategy was aimed at prioritizing patients’ safety, thus avoiding prolonged VT induction in fragile patients with reduced ejection fraction. After EGM acquisition, RFCA was carried out in a power-controlled mode on all the arrhythmogenic targets determined according to standard clinical practice at the time of the procedures (i.e., not always strictly related to local abnormal ventricular activity or late potentials). Finally, a conventional remap and electrophysiological study implementing programmed stimulation were carried out to evaluate substrate modification and VT inducibility. Indeed, the clinical endpoint of the ablation procedure was the non-inducibility of any VT at the end of the protocol.

## Software tools for electrophysiological data annotation and expert consensus

Electrophysiological data annotation was performed using a custom graphical user interface (GUI) entirely developed in MATLAB (Mathworks, Natick, MA, USA) compatible with both Microsoft Windows and MAC OS operating systems [1]. The GUI is a user-friendly tool which is composed of a main window, called Annotation window, encompassing all the necessary features and components to load the electrophysiological data exported by the CARTO 3V6 system, visualize, annotate, and delineate the EGMs. Along with the EGM, the GUI is also able to show the LAT or voltage EA map (which can be manually rotated, and pinpoints, with a black dot, the position on the map corresponding to the presented EGM), and the surface 12-lead ECG. Everything is normally referred to the last beat of the short-term EGM segment, but it is possible to visualize the whole 2.5-s epoch.

The annotators were provided with detailed instructions for using the annotation GUI and were put in the same conditions to perform the annotation, thus promoting consistency. This included a preliminary meeting where they had the chance to discuss the annotation process and the GUI, focusing on general operational aspects, such as the functionality of interface elements, navigation (e.g., zooming), and display features, ensuring consistent and efficient use of the annotation platform.

As regards the expert consensus, it was held remotely, under the guidance of a uniquely designated moderator. The moderator presented every EGM to the annotators, along with the information about the three independent annotations. When an EGM was associated with one or more AVP labels, the GUI allowed inspecting the corresponding delineation onsets and ends. The annotators were asked to discuss the nature of the displayed EGM, and to reach a unanimous decision on the EGM label (i.e., AVP, Physiological, Unknown). If the annotators agreed on assigning the AVP class, they were then prompted to discuss and finalize the AVP delineation. In this latter phase, they had the opportunity to choose from the previously delineated onset and end provided by all the annotators who marked that EGM as an AVP, or to identify a new location for one or both temporal fiducial points. This new GUI (shown in **Fig. S1**) resembled the Annotation GUI [1] except for the lower section of the window, which in this case was optimized for the purpose.

**
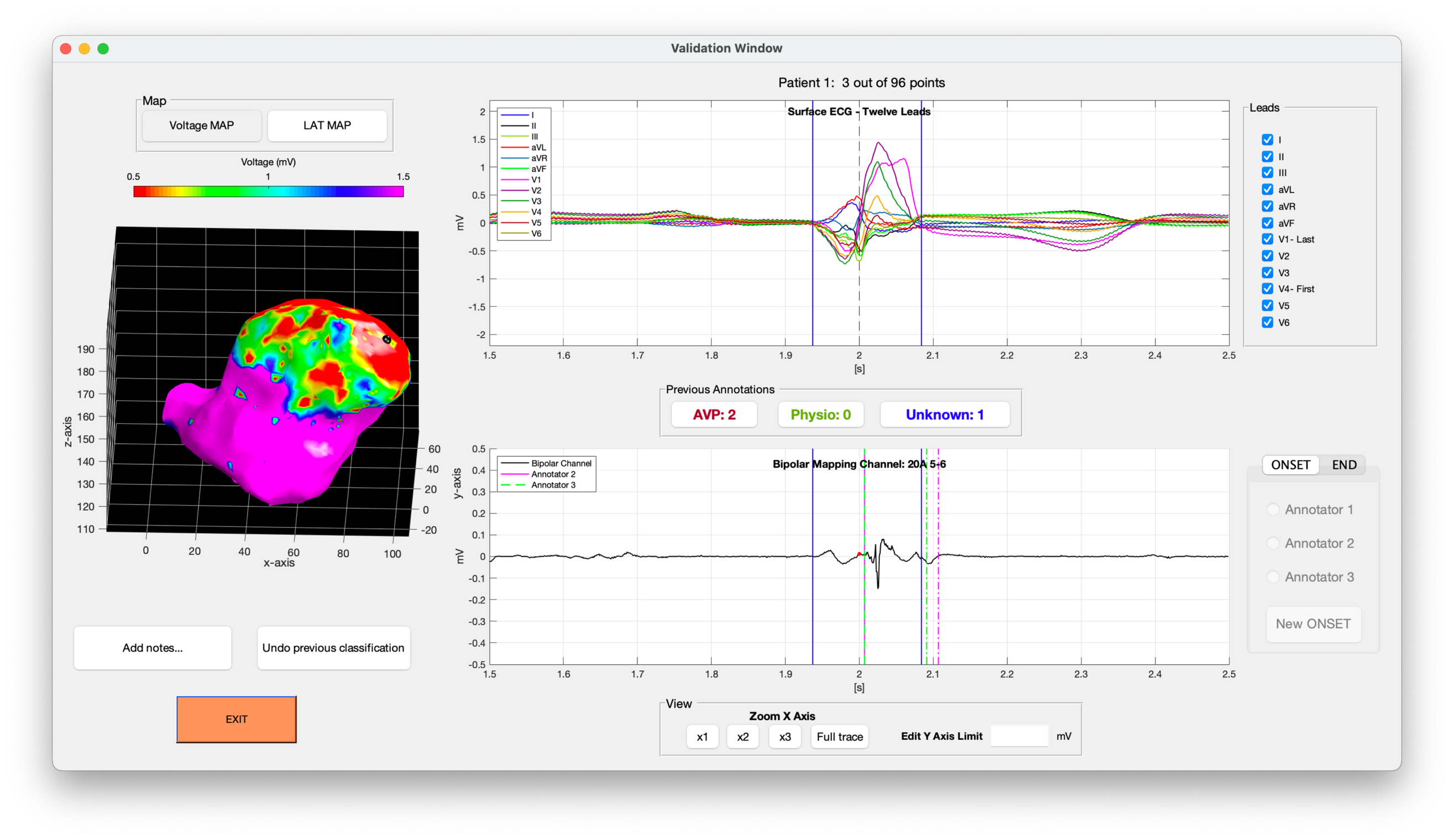
**

**Fig. S1. The GUI used for the expert consensus.** It consists of three main parts: the bipolar voltage/LAT map which the current bipolar EGM refers to (on the left), the 12-lead surface ECG (top) and the bipolar EGM to be discussed (bottom). In the top plot, the two blue vertical lines identify the earliest and latest QRS complex activations on the 12-lead surface ECG. Above the bipolar EGM plot three buttons are displayed, serving as vote count for each class assigned during the independent annotation, and as selection tool for the consensus-driven class. On the right side, buttons allow users to select annotations among those provided by each of the three experts, as well as for new delineation (NEW ONSET/NEW END). To mitigate bias, these annotations are displayed with anonymized color-coded markers (magenta, blue, green). The interface facilitates unanimous decision-making for both class assignment and temporal delineation.

# Data Records

The dataset was hierarchically structured, with a directory assigned to each patient (Pt) out of the nine involved in the study, ensuring a compartmentalized and efficient retrieval mechanism. For each patient, as detailed in **Table S1**, the electrophysiological data, the information on the EA map reconstruction of the LV, and the annotation in terms of class and delineation of the AVP were provided. As the ARGO dataset will be released on Physionet [2], the file formats adhere to the WFDB specifications [3].

Each n-th data point Pn is associated with two files: the data file and the header file. The data file, *Pn.dat*, contains the electrophysiological measurements column-wise, in a 2500x15 matrix, where the columns report the bipolar EGM, the pair of unipolar EGMs, and the 12 leads of the surface ECG (I, II, III, aVL, aVR, aVF, V1, V2, V3, V4, V5, V6). All these signals, sampled at 1 kHz, were stored without additional processing other than the bandpass filtering performed by the CARTO^®^3 mapping system. It is important to note that this data matrix was stored as dimensionless integer values (analog-to-digital converter units). Consequently, to convert these values into amplitude measurements expressed in mV, they needed a multiplication by a gain factor, 0.003 mV, representing the digital data resolution. Supplementing this data file, the *Pn.hea* header, composed of line- and field-oriented ASCII text, is provided, which contains meta-data detailing technical aspects like lead names, analog-to-digital converter gain, number of signals, sampling frequency and number of samples per signal. A comprehensive description of the metadata reported in the header file is shown in **Table S2**. It is noteworthy that in the “Description” field the encoding of both the catheter type and the electrode pair used to obtain each bipolar recording are reported. Specifically, for catheter type the label “M" corresponds to the ThermoCool SmartTouch catheter (e.g., M1-2) and the label “20A_” corresponds to the PentaRay catheter (e.g., 20A_1-2), with the suffix indicating the electrodes involved in the bipolar signal.

Moreover, annotations were provided in distinct WFDB-compatible files for every annotator and for the expert consensus, namely *Pn.annotation_ann1*, *Pn.annotation_ann2*,

Table S1 - A schematic representation of the data files contained within the patient's folder, detailing the electrophysiological and the LV reconstruction data. The table outlines the type of data, file formats, and their specific content.

| **Data** | **File** | **Content** |
| --- | --- | --- |
| **Electrophysiological** | Pn.dat | Electrophysiological recordings (bip, unip1, unip2, 12-lead ECG) |
|  | Pn.hea | Header |
|  | Pn.annotation_ann1 | Annotation and delineation data of Annotator1 |
|  | Pn.annotation_ann2 | Annotation and delineation data of Annotator2 |
|  | Pn.annotation_ann3 | Annotation and delineation data of Annotator3 |
|  | Pn.annotation_consensus | Annotation and delineation data of expert consensus |
| **LV reconstruction** | XYZmesh.txt | 3-D coordinates of the triangulated mesh vertices composing the LV reconstruction |
|  | ConnectivityList.txt | List of connection between triangulated mesh vertices |
|  | MESHcoloring.txt | Voltage and LAT map coloring data |
|  | POS_POINTS.txt | Pn coordinates on the LV reconstruction |
|  | AblationPoints.txt | Coordinates of the ablated points onto the LV map |

Table S2 - A schematic representation of the content of the header file Pn.hea.

| **Header example** | |
| --- | --- |
| P71 15 1000 2500  P71.dat 32 333.3333(0)/mV 0 0 4 370 0 20A_1-2 | |
| **Field** | **Description** |
| P71 | A string of characters that identify the record. |
| 15 1000 2500 | Number of signals, sampling frequency and number of samples per signal |
| P71.dat | File name |
| 32 | Storage format of the signal |
| 333.3333 | Analog-to-digital converter gain ($mV^{-1}$) |
| (0) | Baseline, specifying the sample value corresponding to 0 physical unit |
| /mV | Units |
| 0 | Analog-to-digital converter resolution (default value) |
| 0 | Analog-to-digital converter zero (default value) |
| 4 | The initial value of the signal |
| 370 | Signed checksum of the signal |
| 0 | Block size (default value) |
| 20A_1-2 | Description (i.e., catheter type and electrode pair) |

*Pn.annotation_ann3* and *Pn.annotation_consensus*. These files report the class annotation (i.e. “P”, “A” or “U”), the delineation onset and end of the AVP in terms of sample index, respectively indicated as “(”and “)”. If some annotators did not label the n-th signal as AVP, the corresponding delineation information was reported with single “ " ” with a default out-of-range sample index (i.e., 9999).

Lastly, for the reconstruction of the LV EA map as a triangulated mesh, all required details were reported in text files (.txt); specifically, for each patient, five files were included:

- *XYZmesh.txt*: it reports the vertex list of the mesh. It has been stored as a matrix with three columns, representing the X, Y, and Z coordinates for each vertex.
- *ConnectivityList.txt*: it describes the topological structure of the triangulated mesh vertex connectivity information. The matrix stored in this file is m-by-3, where m denotes the cardinality of triangles of the mesh. The elements in each column are indices and have a direct correspondence to vertex positions in the XYZmesh file.
- *MESHcoloring.txt*: it provides the scalar attributes associated with each vertex of the mesh (per row), such as the voltage and LAT maps. This information allows for an intuitive visualization of voltage or LAT distributions across the mesh.
- *POS_POINTS.txt*: it lists the spatial coordinates where the EGM points are mapped onto the mesh. Each row in this file corresponds to a point in the three-dimensional space, represented by its X, Y, and Z coordinates, and describes the locations where the electrophysiological measurements are projected on the mesh.
- *AblationPoints.txt*: this file contains the spatial coordinates of the map where the ablation has been performed. Each row in this file corresponds to an ablation point in the three-dimensional space (as X, Y, and Z coordinates).

# Usage Notes and Code Availability

The electrophysiological recordings and the related annotation data were formatted using the standard WFDB software package format and can be imported and analyzed using functions such as rdsamp and rdann from the WFDB Software Package28. Remarkably, the same information provided in WDFB format is made available in a unique MATLAB struct variable (i.e., ARGODataset_MATLAB.mat).

Beyond the dataset, a set of custom MATLAB functions (The MathWorks, MA, USA) has been developed to facilitate the reading and visualization of both electrophysiological and electroanatomical data. Specifically, the first function (VisualizeRecordingsARGO.p) enables the simultaneous display of 12-lead ECG recordings, the bipolar electrogram, and corresponding unipolar leads, along with delineation markers provided by expert annotators and the final consensus (in case of AVP only). This visualization occurs in a separate window, allowing for detailed inspection. Additionally, a GUI (VisualizeMapsARGO_GUI.p) has been implemented and provided to the interested reader to visualize electroanatomical maps, including all routinely acquired maps during such electrophysiological studies, i.e., voltage and LAT maps, along with all targeted sites for ablation during the clinical procedure. These tools enable efficient browsing of both electrophysiological and electroanatomical data.

# Validation results

## Signal morphologies and substrate characterization

To provide a clearer visual overview of the signal morphologies represented in the ARGO dataset, **Fig. S2** reports representative examples of bipolar EGMs belonging to the three annotated classes, namely Physiological EGMs, abnormal ventricular potentials (AVPs), and Unknown EGMs. These examples are intended to qualitatively illustrate the main differences among the signal types included in the dataset.

Moreover, a descriptive characterization of the mapped ventricular tissue was performed based on bipolar EGM peak-to-peak amplitude values, to provide additional information on the LV substrate represented in ARGO dataset. Because the provided EA maps are presented as reconstructed chamber geometries with voltage information interpolated by the CARTO system from the acquired mapping points, substrate characterization was performed on the interpolated bipolar voltage values associated with the chamber representation, rather than directly on the acquired points alone. This choice was made to remain consistent with the graphical voltage representation used during the clinical procedure and with the map representation provided in the dataset.


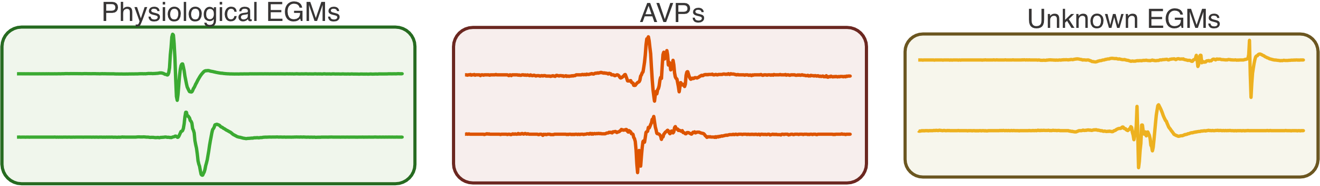


**Fig. S2. Examples of bipolar EGMs across the annotated classes.** Representative examples of bipolar EGMs belonging to the three annotated classes included in the ARGO dataset: Physiological EGMs, AVPs, and Unknown EGMs.

Following commonly adopted voltage ranges in substrate-guided VT mapping, the interpolated bipolar amplitudes were grouped into three voltage-defined substrate classes:

- Dense scar: bipolar amplitude < 0.5 mV
- Border zone: bipolar amplitude between 0.5 and 1.5 mV
- Healthy tissue: bipolar amplitude > 1.5 mV

For each patient, the proportion of mapping points belonging to the three voltage ranges was computed and expressed as percentage of the total available points. **Fig. S3** reports the resulting distribution of voltage-defined substrate classes across the nine patients included in the ARGO dataset.

Dense scar areas, defined by bipolar amplitudes below 0.5 mV, account for a smaller but clearly identifiable portion of the mapped myocardium, reflecting the heterogeneous structural remodeling typically observed in post-ischemic ventricular substrates.


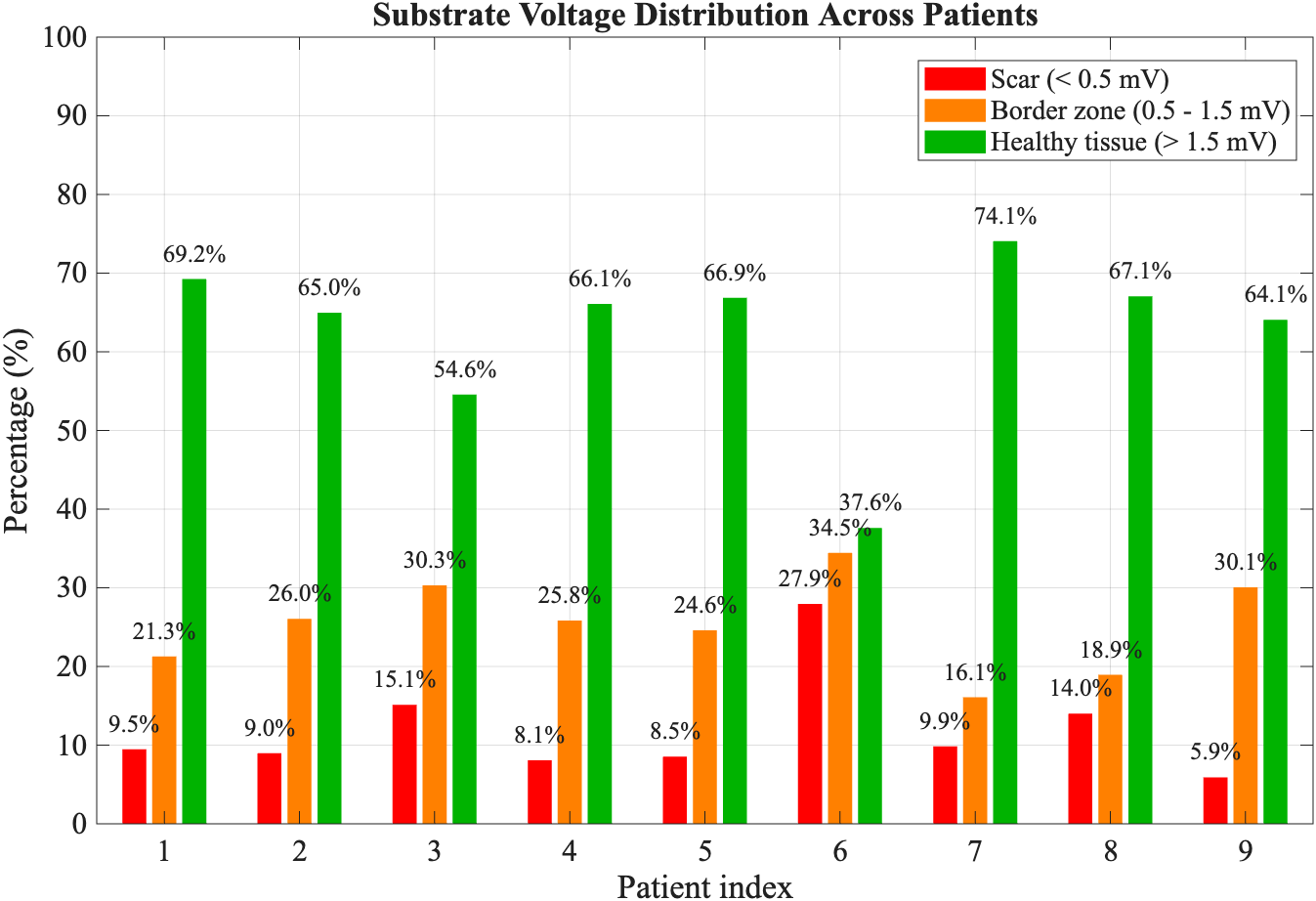


**Fig. S3. Substrate voltage distribution across patients.** Percentage distribution of substrate voltage classes across the nine patients included in the dataset. For each patient, the proportion of mapping points in dense scar (<0.5 mV), border zone (0.5-1.5 mV), and heathy tissue (>1.5 mV) is reported according to standard bipolar voltage threshold used in clinical practice.

## Delineation examples

**Fig. S4** reports representative examples of AVP onset and end annotations obtained during the final consensus procedure. These examples highlight the variability that AVP morphologies can exhibit, including differences in amplitude, fragmentation, and temporal appearance.


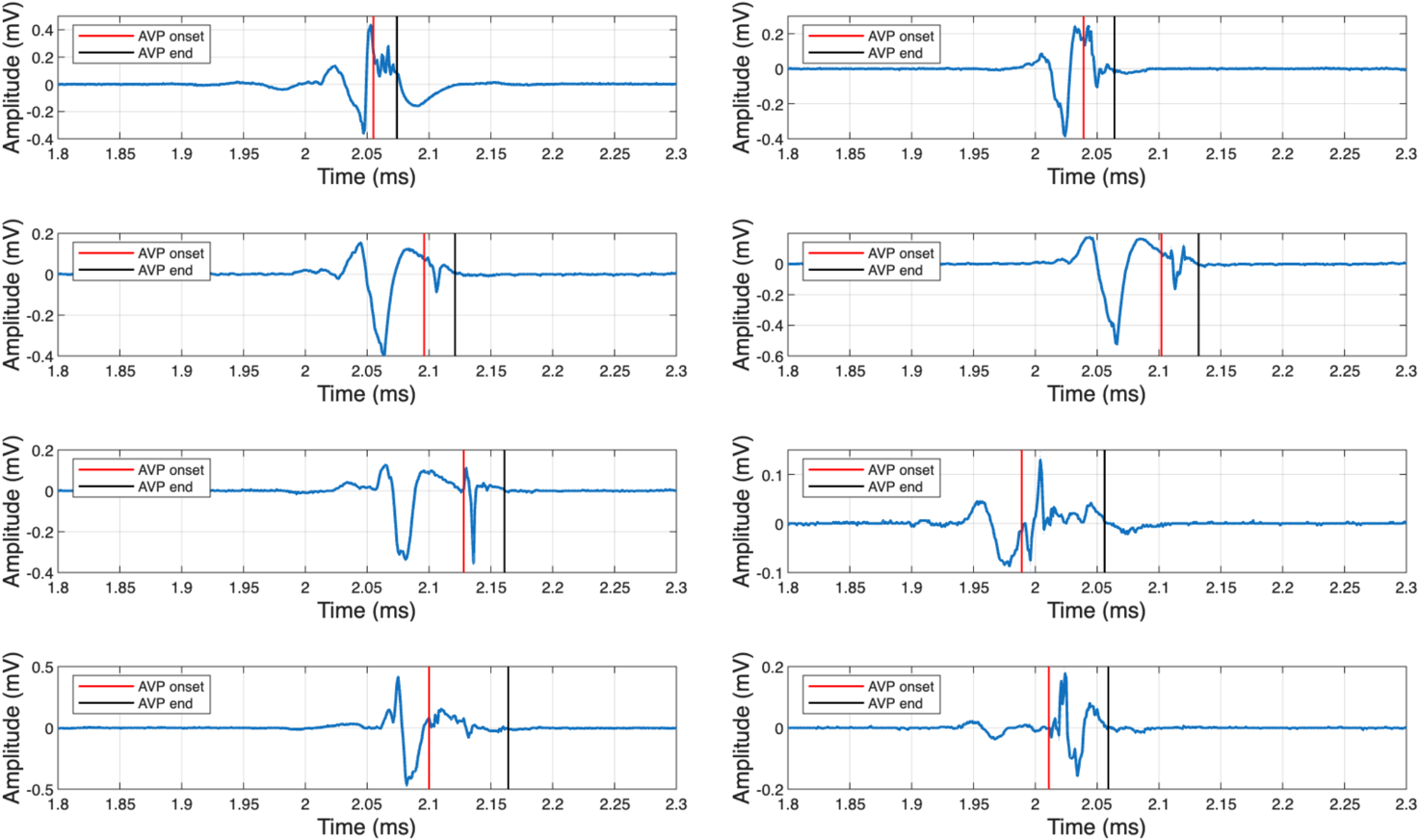


**Fig. S4. Examples of AVP delineations.** Representative examples of AVP delineations from the final consensus. In each EGM, the red and black vertical markers indicate the annotated AVP onset and end.

## Annotation disagreement

As illustrated by the representative EGM examples reported in **Fig. S1**, the morphologies of these signals can be highly heterogeneous and complex, making the processes of recognition and annotation inherently challenging. This aspect becomes evident when examining signals that generated disagreement during the annotation and consensus procedures. In particular, **Fig. S5** reports representative examples of EGMs that generated disagreement between Annotator 1 and the final consensus annotation. According to the validation analysis presented in the main manuscript, Annotator 1 showed the highest level of disagreement with respect to the consensus (Cohen’s κ = 0.58), while Annotator 2 and Annotator 3 exhibited higher agreement levels (κ = 0.76 and κ = 0.78, respectively).

These examples therefore illustrate that the observed annotation variability does not originate from random inconsistencies but rather from the intrinsic ambiguity of certain EGM morphologies. Indeed, **Fig. S5** illustrates how far-field interference and low-amplitude deflections may act as primary sources of disagreement.

Such findings further confirm that a single-expert evaluation is insufficient for complex EGM signals, justifying the necessity of the rigorous, multi-expert consensus process used to establish the ARGO ground truth, allowing discussion of ambiguous cases and the definition of a final shared annotation.


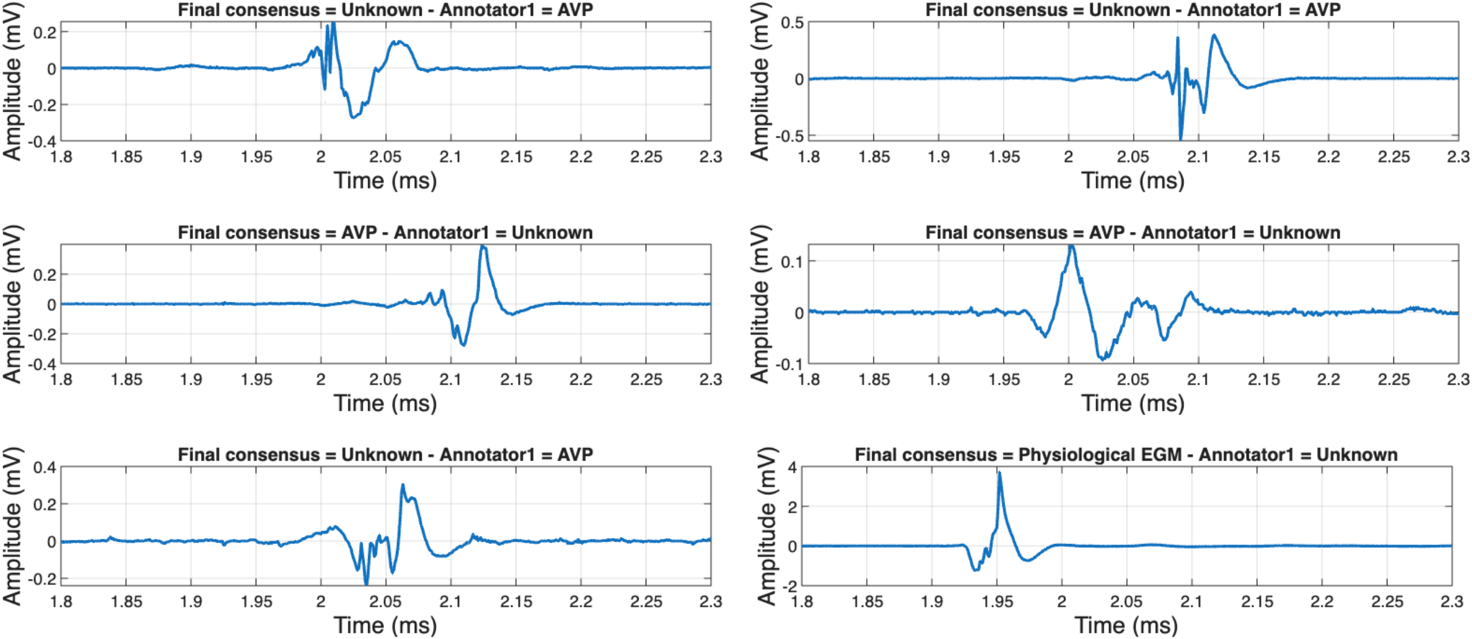


**Fig. S5. Examples of annotation disagreement.** Representative examples of EGMs that generated disagreement between Annotator1 and the final consensus annotation.

# Bibliography

[1] M. Orrù, E. Pitzalis, G. Baldazzi, G. Viola, and D. Pani, “A novel tool for the annotation of bipolar electrograms for cardiac electrophysiology,” in *8th National Congress of Bioengineering, GNB 2023*, Patron Editore S.r.l., 2023.

[2] A. L. Goldberger *et al.*, “PhysioBank, PhysioToolkit, and PhysioNet: components of a new research resource for complex physiologic signals,” *Circulation*, vol. 101, no. 23, pp. e215–e220, 2000.

[3] I. Silva and G. B. Moody, “An open-source toolbox for analysing and processing physionet databases in matlab and octave,” *J. Open Res. Softw.*, vol. 2, no. 1, 2014.
